# Effect of intravitreal injections due to neovascular age-related macular degeneration on retinal nerve fiber layer thickness and minimum rim width: a cross sectional study

**DOI:** 10.1186/s12886-024-03453-2

**Published:** 2024-04-23

**Authors:** Agnes Boltz, Tanja Spöttl, Wolfgang Huf, Birgit Weingessel, Veronika Pia Vécsei-Marlovits

**Affiliations:** 1 Department of Ophthalmology, Hietzing Hospital, Wolkersbergenstraße 1, Vienna, 1130 Austria; 2 Karl-Landsteiner Institute for Process Optimization and Quality Management in Cataract Surgery, Vienna, Austria; 3 Department of Laboratory Medicine, Hietzing Hospital, Vienna, Austria; 4 Karl Landsteiner Institute for Clinical Risk Management, Vienna, Austria

**Keywords:** Retinal nerve fiber layer thickness, Minimum rim width, Intravitreal injections, Anti-VEGF, CNV

## Abstract

**Purpose:**

The present study tested the hypothesis that repeated anti-VEGF injections are associated with reduced retinal nerve fiber layer (RNFL) and minimum rim width (MRW) of the optic nerve head.

**Patients and methods:**

Sixty-six patients with a history of intravitreal injections due to neovascular age-related macular degeneration were included. RNFL and MRW were measured using optical coherence tomography (Spectralis OCT, Heidelberg Engineering, Heidelberg, Germany).

**Results:**

Mean global RNFL was 90.62 μm and both RNFL as well as MRW significantly decreased with advanced age (*p* = 0.005 and *p* = 0.019, respectively). Correlating for the number of injections, no significant impact on RNFL was found globally (*p* = 0.642) or in any of the sectors. In contrast, however, global MRW was significantly reduced with increasing numbers of intravitreal injections (*p* = 0.012). The same holds true when adjusted for the confounding factor age (RNFL *p* = 0.566 and MRW *p* = 0.023).

**Conclusion:**

Our study shows that repeated intravitreal injections due to choroidal neovascularization seem to have a deleterious effect on MRW but not on RNFL. This suggests that MRW is a more sensitive marker than RNFL for evaluating the effect of frequent intravitreal injections on the optic nerve head since it seems to be the first structure affected.

## Background

Around 15 years ago, the introduction and utilization of intravitreal injections of anti-vascular endothelial growth factor (anti-VEGF) antibodies has tremendously enhanced the treatment options and visual outcome for several eye diseases associated with macular edema, such as neovascular age-related macular degeneration (n-AMD), retinal vein occlusion, and diabetic retinopathy [1–9]. In general, anti-VEGF injections are considered safe and the benefits outweigh by far the possible ocular complications [10]. However, unlike laser therapy, patients usually have to undergo frequent reinjections each year to remain visually and morphologically stable. Subsequently, the risk of adverse events may be increased by the continuous long-term. As one would assume, the loading of additional fluid into the eyeball can raise intraocular pressure (IOP). Therefore, several studies were conducted investigating IOP following anti-VEGF injections [11–13]. A meta-analysis of 46 studies on this topic showed that IOP was significantly increased for all measured time-intervals of the day of injection, but slightly decreased on the day after injection and returned to normal thereafter [14]. These IOP fluctuations along with possible fluctuations in blood perfusion of the optic nerve head may lead to glaucoma over time.

The introduction of Optical Coherence Tomography (OCT) made it possible to detect early structural changes and at the same time deliver objective parameters in contrast to perimetry. In addition to a thinning of peripapillary retinal nerve fiber layer thickness (RNFL), another OCT parameter, i.e. Bruch’s membrane opening minimum rim width (MRW) has been established more recently for the assessment of optic discs. Hence, the present study tested the hypothesis that repeated anti-VEGF injections are associated with reduced RNFL and MRW of the optic nerve head.

## Materials and methods

**Fig. 1:**
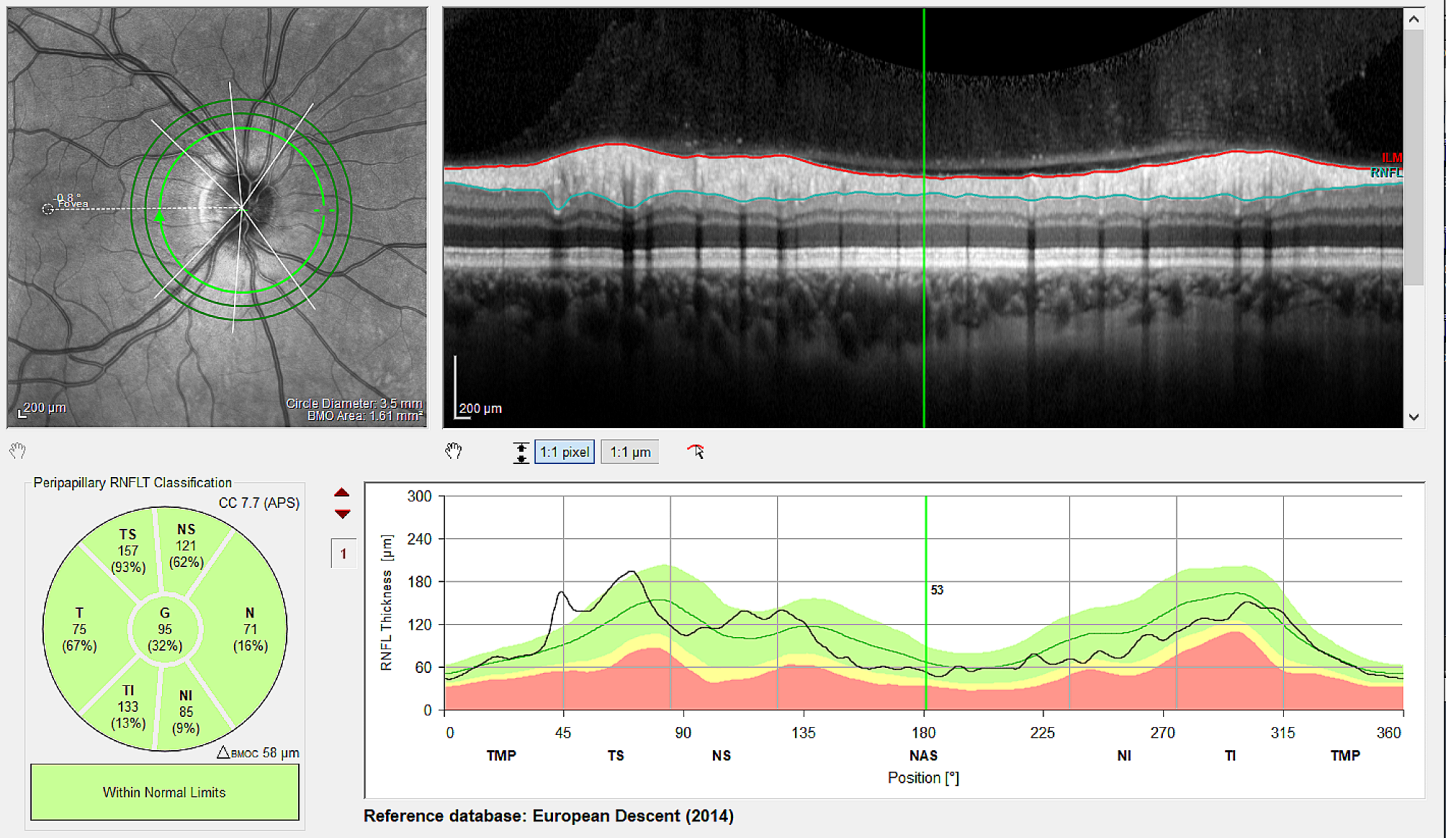
Exemplary retinal nerve fiber layer (RNFL) measurement

**Fig. 2:**
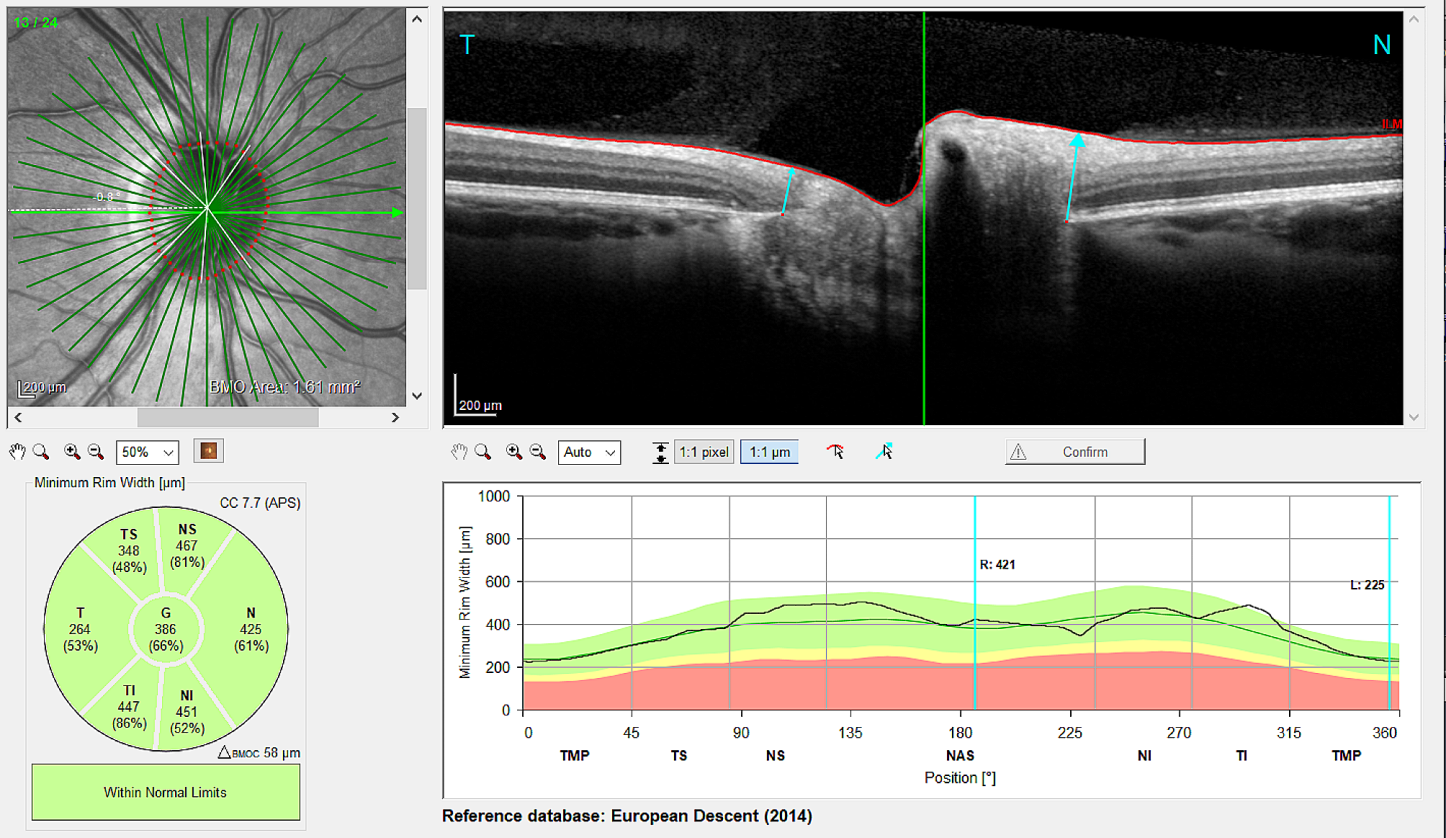
Exemplary minimum rim width (MRW) measurement

This cross sectional study was conducted after approval from the Ethics Committee of the City of Vienna had been obtained (EK 20-352-VK) and adhered to the tenets of the Declaration of Helsinki. Due to the retrospective character of the study, informed consent was waived in agreement with the positive vote of the above-mentioned Ethics Committee. Patients with a history of intravitreal injections due to neovascular AMD were included. Intravitreal injections with anti-VEGF were applied without paracentesis and according to the pro-re-nata regimen. RNFL and MRW were measured using optical coherence tomography (Spectralis OCT, Heidelberg Engineering, Heidelberg, Germany) and analyzed using the build in software (Heyex 2, Figs. 1 and 2) with included age-matched reference values. If necessary, manual corrections of retinal nerve fiber layer segmentation and Bruch’s membrane opening were undertaken by a trained physician. Exclusion criteria were presence of uncontrolled elevated IOP over 21mmHg prior to injections, a history of ischemic opticus atrophy, diagnosis of glaucoma prior to anti-VEGF treatment as well as presence of a clinically significant epiretinal membrane and high myopia. Statistical analysis and linear regression models were carried out in R (version 4.0.3, R Foundation for Statistical Computing, Vienna, Austria).

## Results

**Fig. 3:**
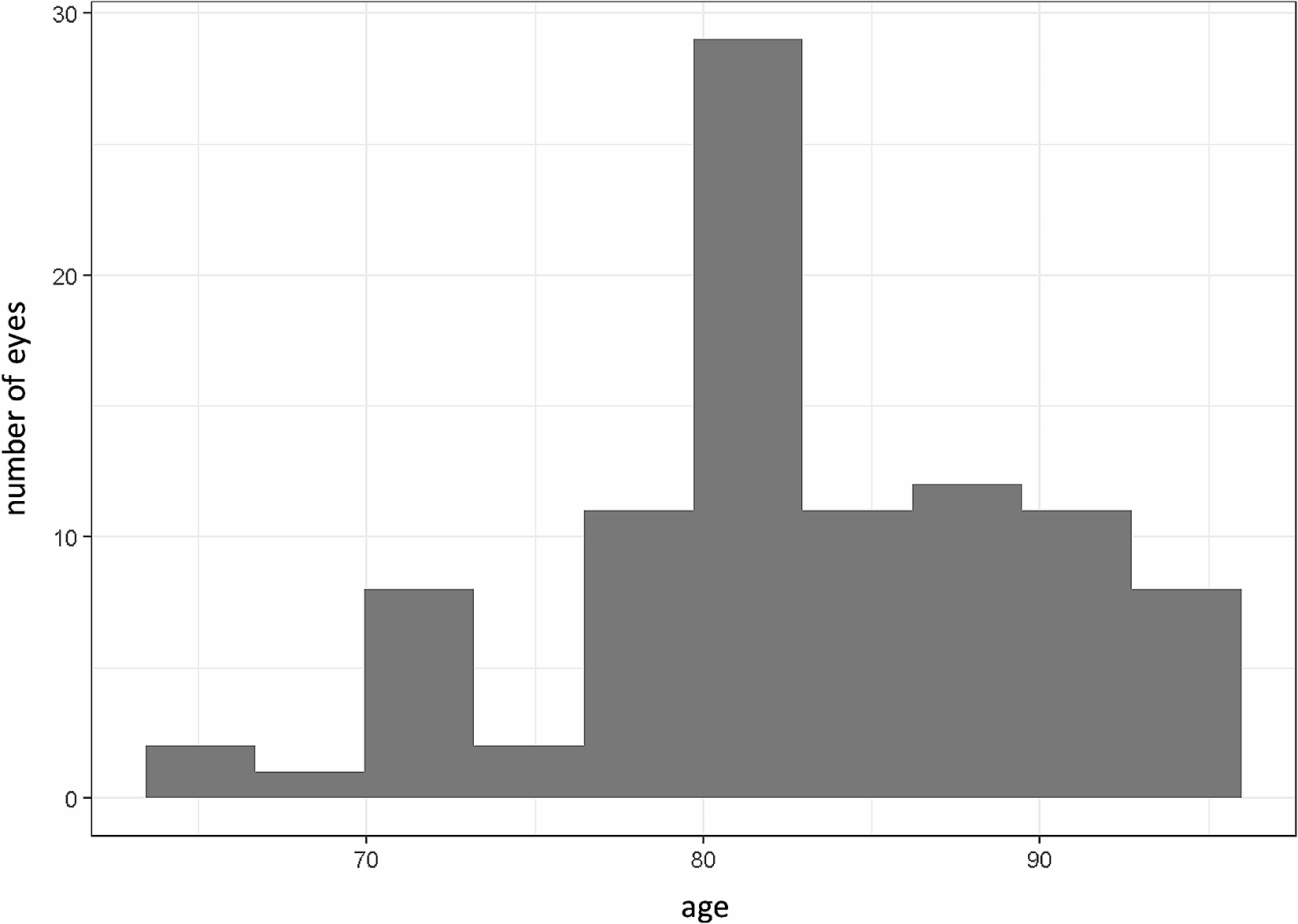
Age distribution and number of eyes

Sixty-six eyes of patients with CNV and a mean age of 83.4 years and an average of 12.58 prior injections were included (Fig. 3).

**Fig. 4:**
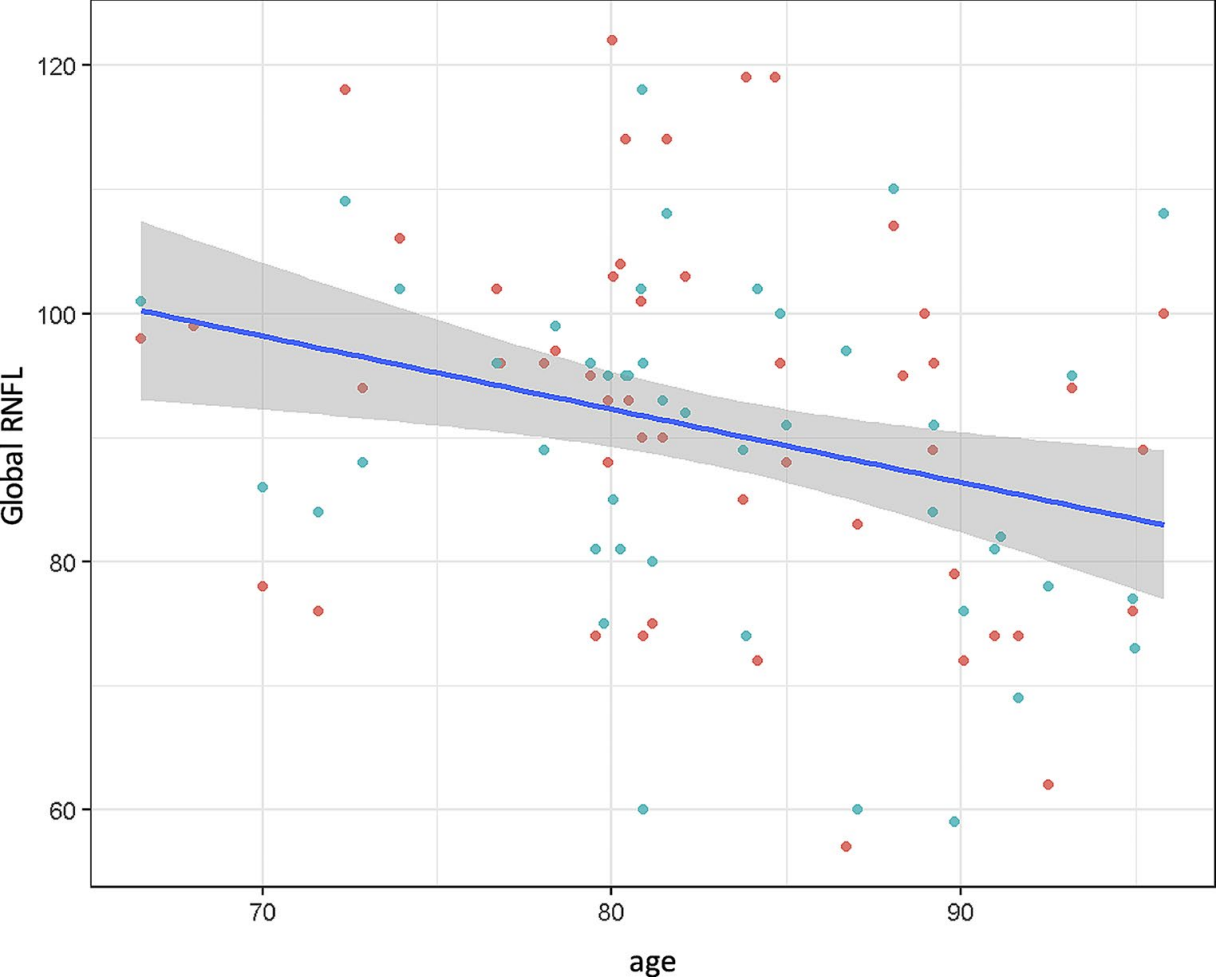
Global retinal nerve fiber layer (RNFL) by age. Green dots represent right eyes, red dots represent left eyes; *p* = 0.005

Mean global RNFL was 90.62 μm and significantly decreased with advanced age (*p* = 0.005, Fig. 4).

**Fig. 5:**
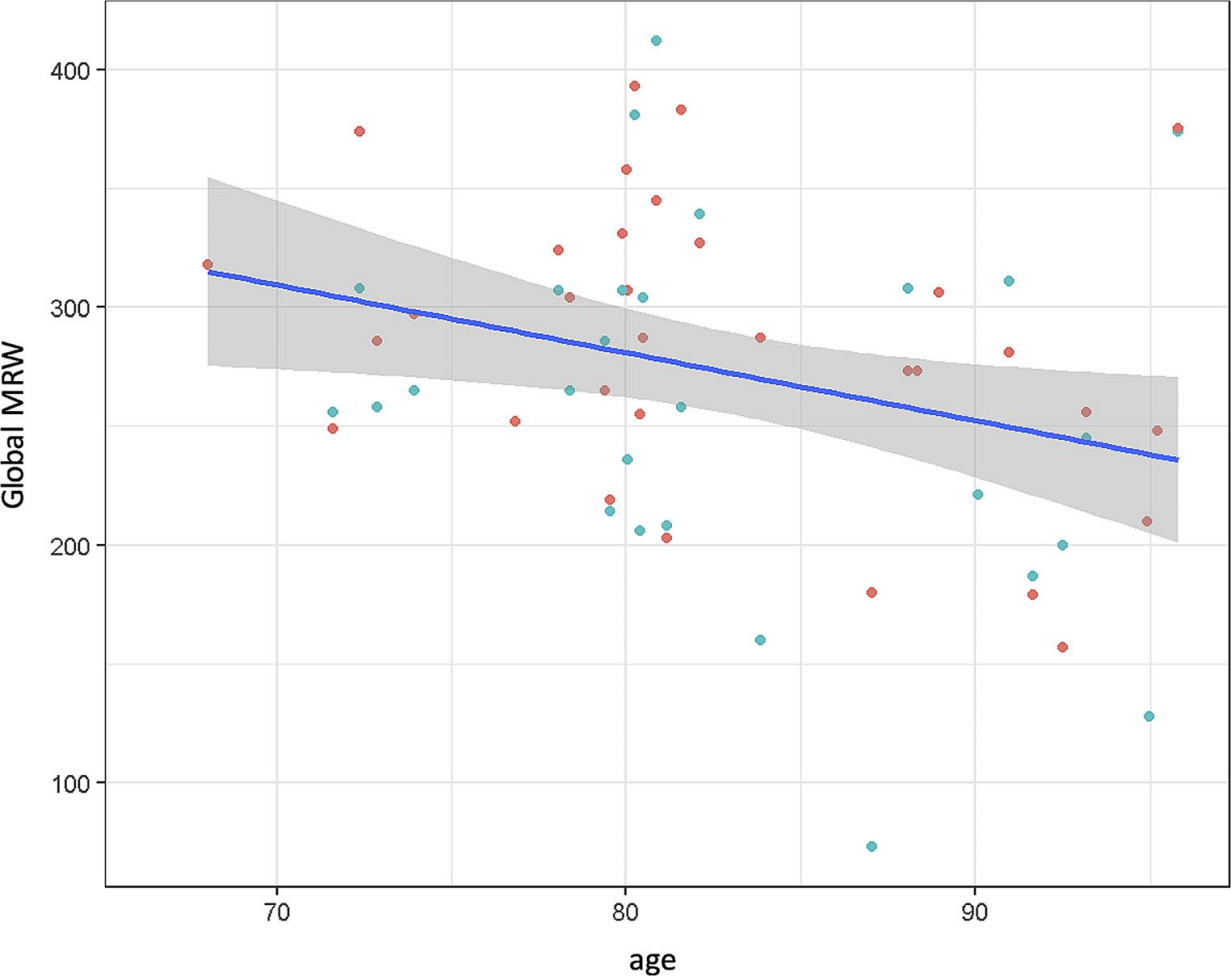
Global minimum rim width (MRW) by age. Green dots represent right eyes, red dots represent left eyes; *p* = 0.019

Similarly, RNFL of the nasal-superior (*p* = 0.025), temporal-superior (*p* < 0.001), temporal-inferior (*p* = 0.004) sectors highly significantly correlated with age as well, whereas this did not hold true for the nasal (*p* = 0.237), the temporal (*p* = 0.216), and the nasal-inferior (*p* = 0.862) sector. Advanced age was also significantly associated with lower global MRW (*p* = 0.019, Fig. 5), nasal-superior (*p* = 0.020), temporal-superior (*p* = 0.014), temporal (*p* = 0.004), and temporal-inferior (*p* = 0.019) MRW, but not with nasal (*p* = 0.272) and nasal-inferior (*p* = 0.103) MRW.

**Fig. 6:**
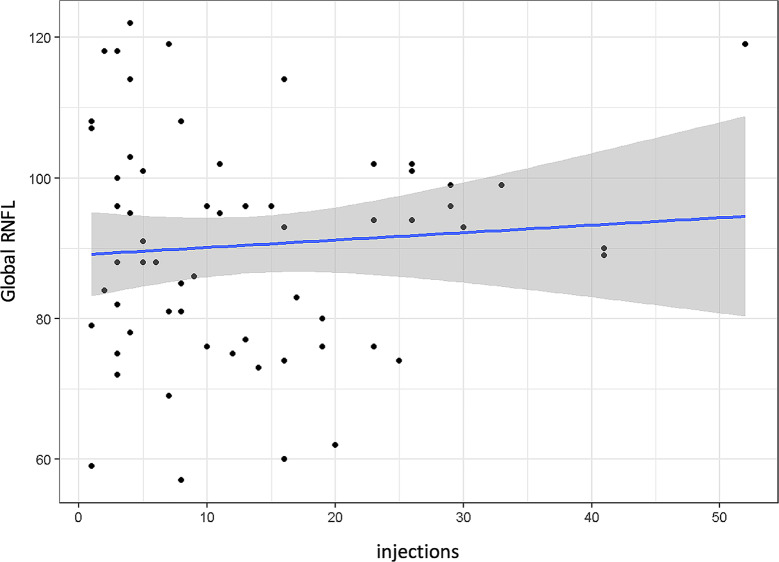
Global retinal nerve fiber layer (RNFL) by total number of intravitreal injections; *p* = 0.642

Correlating for the number of injections, no significant impact on RNFL was found globally (*p* = 0.642, Fig. 6) or in any of the sectors.

**Fig. 7:**
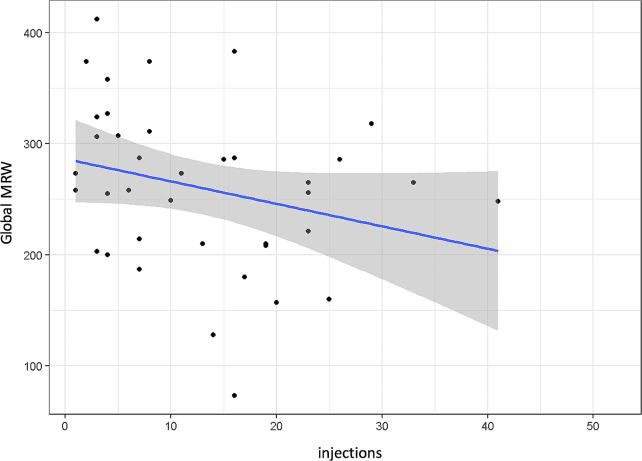
Global minimum rim width (MRW) by total number of intravitreal injections; *p* = 0.012

In contrast, however, global MRW was significantly reduced with increasing number of intravitreal injections (*p* = 0.012, Fig. 7).

**Fig. 8:**
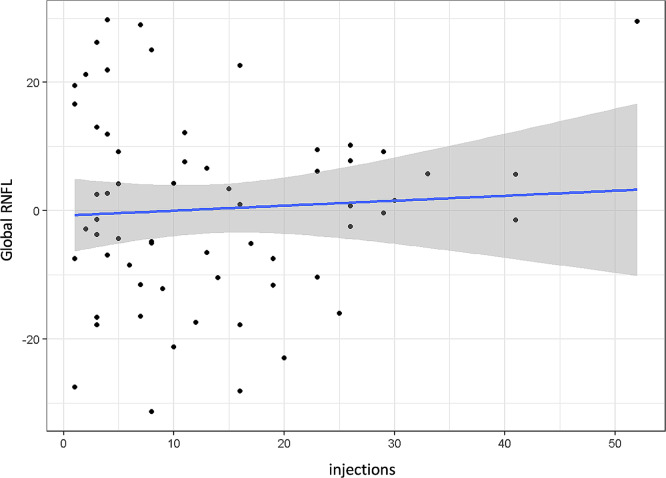
Global retinal nerve fiber layer (RNFL) adjusted for age (residuals) and grouped by total number of intravitreal injections; *p* = 0.566

**Fig. 9:**
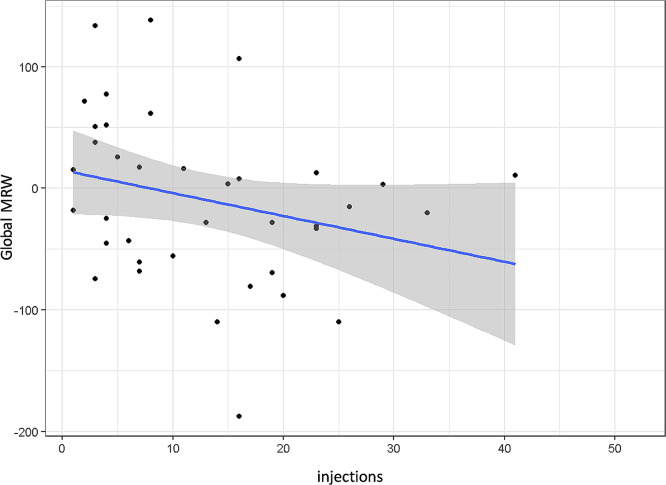
Global minimum rim width (MRW) adjusted for age (residuals) and grouped by total number of intravitreal injections; *p* = 0.023

Adjusting for the confounding factor age, the level of significance neither changed for RNFL (*p* = 0.566, Fig. 8) nor for MRW (*p* = 0.023, Fig. 9).

Looking at the MRW sectors individually, the temporal (*p* = 0.011), and the temporal-inferior (*p* = 0.044) sector also significantly correlated with number of injections, whereas the other sectors did not (nasal *p* = 0.079; nasal-superior *p* = 0.099; temporal-superior *p* = 0.065; nasal-inferior *p* = 0.141).

## Discussion

Our study shows that repeated intravitreal injections due to choroidal neovascularization seem to have a deleterious effect on MRW but not on RNFL. The latter is in accordance with the majority of previous studies conducted on this topic [14–22]. However, two studies by Parlak et al. and Martinez-de-la-Casa et al. found a significant thinning of RNFL after administration of ranibizumab in patients with CNV [23, 24]. The former study found a significant reduction in RNFL in both, the treatment arm as well as the untreated control arm with dry AMD at follow up, but no significant difference between both groups which questions the hypothesis that anti-VEGF injections caused this effect. The latter study only included treatment naïve patients, and as such they showed increased macular thickness, especially in the nasal quadrant, which was significantly reduced after treatment. One can speculate that the reduction of fluid in the nasal macula may also have an impact on RNFL measurements especially given that most reduction happened in the temporal sector of the optic nerve head as well as at first follow-up at 3 months and did not significantly change thereafter over the course of 12 months [24]. This effect may also explain the findings of another study that associated a thickening on the temporal RNFL quadrant to repeated anti-VEGF injections [25].

Limitations of our own study are the lack of axial eye length measurements which may have an effect on MRW analysis. We tried to minimize this by excluding patients with high myopia.

The lack of effect on RNFL in our study may be attributed to the average number of injections of 12.58 prior to inclusion, and RNFL thinning possibly presents itself just after a longer period of treatment which may also explain the results of the afore mentioned studies mainly in treatment naïve patients. Whether this can only be seen after a higher number of injections (> 30 and more) as suggested by a cross-sectional paper [26] remains to be seen in further studies.

To the best of our knowledge, only one other study investigated the effect of repeated intravitreal injections on other biomarkers of the optic nerve head, such as Bruch’s membrane opening (BMO) [27]. In the study conducted in 29 patients with CNV, diabetic edema, and retinal vein occlusion, a significant increase in BMO was found immediately, i.e. 5 min after each of the first 3 anti-VEGF injections, but this effect did not seem to persist after 12 months. In accordance with our paper, they found no negative effect on RNFL.

The mechanisms underlying the different response of MRW and RNFL to anti-VEGF injections are not clearly understood. However, recent papers have suggested that the rate of change in MRW is significantly greater than RNFL in patients with glaucoma over the course of disease and thus per se a more sensitive biomarker [28, 29]. 

Since IOP fluctuations after intravitreal injections are only temporary, one also needs to take other potential pathways of optic nerve head damage into account. As such, blood perfusion has been shown to play a critical role in the pathogenesis of glaucoma [30]. VEGF can induce the release of nitric oxide [31] and thereby improve blood flow. Another mechanism may be that VEGF seems to have a neuroprotective effect [32, 33]. Therefore, VEGF inhibition potentially leads to both lower perfusion of the optic nerve had and to limited neuroprotection.

## Conclusion

In conclusion, our study suggests that MRW is a more sensitive marker than RNFL for evaluating the effect of frequent intravitreal injections on the optic nerve head since it seems to be the first structure affected. However, further longitudinal studies are warranted to widen our understanding of the potential role of anti-VEGF injections in the pathogenesis of glaucoma.

## Data Availability

The datasets used and/or analysed during the current study are available from the corresponding author on reasonable request.
